# RFX1: a promising therapeutic arsenal against cancer

**DOI:** 10.1186/s12935-021-01952-6

**Published:** 2021-05-08

**Authors:** Joby Issac, Pooja S. Raveendran, Ani V. Das

**Affiliations:** Cancer Research Program, Rajiv Gandhi Centre for Biotechnology, Thycaud.P.O, Thiruvananthapuram, 695014 Kerala India

**Keywords:** Regulatory factor X1 (RFX1), Transcription factor, Cancer cell hallmarks, Tumor suppressor

## Abstract

Regulatory factor X1 (RFX1) is an evolutionary conserved transcriptional factor that influences a wide range of cellular processes such as cell cycle, cell proliferation, differentiation, and apoptosis, by regulating a number of target genes that are involved in such processes. On a closer look, these target genes also play a key role in tumorigenesis and associated events. Such observations paved the way for further studies evaluating the role of RFX1 in cancer. These studies were indispensable due to the failure of conventional chemotherapeutic drugs to target key cellular hallmarks such as cancer stemness, cellular plasticity, enhanced drug efflux, de-regulated DNA repair machinery, and altered pathways evading apoptosis. In this review, we compile significant evidence for the tumor-suppressive activities of RFX1 while also analyzing its oncogenic potential in some cancers. RFX1 induction decreased cellular proliferation, modulated the immune system, induced apoptosis, reduced chemoresistance, and sensitized cancer stem cells for chemotherapy. Thus, our review discusses the pleiotropic function of RFX1 in multitudinous gene regulations, decisive protein–protein interactions, and also its role in regulating key cell signaling events in cancer. Elucidation of these regulatory mechanisms can be further utilized for RFX1 targeted therapy.

## Background

Regulatory factor X1 (RFX1) is a context-dependent transcription factor that can regulate gene expression differently based on the target gene but independent of cellular habitat [1]. Targeting RFX1 is beneficial in many cancers as it counters the possible mechanisms of cancer cell survival and maintenance [2–4]. Cancer cells are difficult to target as they exploit key processes like cell proliferation, differentiation, and apoptosis by modulating the expression of key molecules involved in the regulation of such activities [5]. These attributes help the cancer cells to showcase cellular plasticity, invasion, metastasis, drug resistance, immune evasion, and enter dormancy leading to cancer recurrence [6]. RFX1 reduces cancer cell proliferation by targeting FGF1 [7] and transforming growth factor beta 2 (TGFβ2) [8], induce differentiation by targeting CD44 [2] and c-Myc [9], and promote apoptosis by regulating myeloid cell leukemia sequence 1 protein (MCL1) [10]. RFX1, via targeting these abruptly expressed target genes could possess potential tumor suppressor activity (Table 1).

**Table 1:** Summary of RFX1 targets showing the pleiotropic nature of the RFX1 transcription factor

Target gene/Protein	Target gene/Protein function	RFX1 mediated regulation	Outcome	Refs.
Id2	Repression of helix-loop-helix transcription factors		Rapid increase in Id2 expression upon growth serum stimulation in the NIH3T3 mouse embryo fibroblast cell line	[1]
HBV gene	Viral replication		Induces HBV gene enhancer activity, especially in liver	[36]
MHC class II genes	Antigen presentation; Adaptive immunity; Self-tolerance			[11, 12]
ITGA6	Testis cord development		Critical for proper spermatogenesis in mouse	[46]
MCP1	Monocyte and basophil chemotactic		RFX1 regulated MCP1 is a potential therapeutic strategy for CAD management	[68]
CD70	CD27 ligand; Co-stimulatory signaling		RFX1 increased H3K9 tri-methylation	[153]
ITGAL / CD11a	Cell adhesion; Co-stimulatory signaling		RFX1 increased H3K9 tri-methylation	[153]
TLR4	Activation of innate immune system		RFX1 can regulate innate immunity response and is a potential target for CAD management	[14]
FGF1	Cell proliferation; Neurogenesis		RFX1 disrupts senescence of glioblastoma stem cells by targeting FGF1	[7]
TGF-β2	Wide variety of roles from cell proliferation, motility, differentiation and apoptosis		RFX1 suppresses TGFβ2-ERK signaling pathway and resultant cell proliferation in neuroblastoma cell lines	[139]
COL1A1	Type I collagen fiber		RFX1 binds to methylated COL1A1 near transcription start site	[154]
COL1A2	Type I collagen fiber		RFX1/ MDBP complex binds to the methylated first exon of COL1A2 promoter and recruits HDAC1 and mSin3 enzymes	[64, 154, 155]
CDX2	Intestinal cell growth and differentiation		Rfx1 downregulation coupled with CDX2 overexpression is key in adenocarcinoma tumor progression	[3]
c-Abl	Signal transducer; Proto-oncogene		RFX1 directly interacts and potentiates c-Abl kinase activity	[56]
CD44	Cell surface receptor		RFX1 prevents metastasis of multiple glioblastoma cell lines	[2]
IL-17A	Pro-inflammatory cytokine		RFX1 increased H3K9 tri-methylation and decreased H3 acetylation at IL17A promoter	[15]
RFX1	Context dependent transcription factor		Auto-repression of RFX1 in response to DNA damage	[86]
c-Myc	DNA binding protein; Proto-oncogene;		RFX1 mediated c-Myc downregulation was observed in differentiated HL60 cell line human leukemia cell line	[9]
PCNA	Involved in DNA replication and repair pathways			[156, 157]
IL5RA	Receptor for IL5			[16]
*PTPN6/SHP1*	Cell signaling, differentiation		RFX1/AP4 mediated SHP-1 regulation reduced MCF-7 cell proliferation	[81]
MCL1	Anti-apoptotic factor		RFX1/SHP-1/STAT3/MCL1 axis of autophagy in HCC cells	[10]
EAAT3	Glutamate uptake		RFX1 upregulates EAAT3 in rat neurons and could have treatment applications in neurological disorders	[158]
SPATA4	Spermatogenesis; Modulates cell proliferation, differentiation and apoptosis in many cells		Downregulate Sertoli cell proliferation	[159]
PNRC	Nuclear receptor co-activator			[160]
rpL30	Ribosomal protein			[139]
DNAAF4/DYX1C1	Neuronal migration		RFX1 regulates one of the most replicated candidate genes of Ciliary dyslexia	[161]
DCDC2	Neuronal migration		RFX1 regulates one of the most replicated candidate genes of Ciliary dyslexia	[161]

: Down regulation

: Upregulation

*Id2* inhibitor of DNA binding 2, *HBV* human hepatitis B virus enhancer I, *ITGA6* integrin alpha-6, *MCP1* Monocyte chemoattractant protein, *CAD* Coronary artery disease, *CD70* Cluster of Differentiation 70, *ITGAL / CD11a* integrin subunit alpha L, *FGF1* fibroblast growth factor 1, *COL1A* Collagen α1(I) Gene, *COL1A2* collagen type I alpha 2, *CDX2* Caudal type homeobox 2, *PTPN6*/*SHP1* protein tyrosine phosphatase non-receptor type 6/ Src homology region 2 domain-containing phosphatase-1, *PCNA* proliferating cell nuclear antigen, *EAAT3* Excitatory amino acid transporter type 3, *SPATA4* spermatogenesis associated 4, *PNRC* Proline-rich Nuclear Receptor Coactivator, *rpL30* ribosomal protein *L30*, *DNAAF4/ DYX1C1* dynein axonemal assembly factor 4/ dyslexia 1 candidate 1 gene, *DCDC2* doublecortin domain-containing 2 gene

Amid these, the key role of RFX1 can be attributed to its role in immune system regulation. RFX1 can increase major histocompatibility complex(MHC) class II gene expression [11, 12], key in antigen presentation and cancer cell apoptosis by effector immune cells [13]. From studies relating to cancer and inflammation, it was understood that cancer cells utilize inflammatory signals and pathways for their benefits. RFX1 downregulates such pro-tumor factors like Toll-like receptor 4 (TLR4) [14], Interleukin 17A (IL17A) [15], interleukin 5 receptor subunit alpha (IL5RA) [16], and helps in cancer mitigation while being anti-inflammatory. Another obstacle in cancer treatment has arisen due to the cellular plasticity of the cancer cells. Cancer cells tend to de-differentiate to a more stable cancer stem cell up-on need. Cancer stem cells (CSCs) are a much-more resilient population with increased drug resistance and have been instrumental in cancer recurrence [17].

Targeting CSCs, along with cancer cells, is fundamental to long-lasting treatment efficiency. In this regard, RFX1 tends to induce differentiation of such stem cells and makes them susceptible to conventional therapy. A major hurdle in the current cancer therapy is the increased drug resistance and cancer recurrence, mostly due to the presence of resilient CSCs populations [5, 6]. The ability of RFX1 to induce differentiation of stem cells is seen in normal tissues too. Daf-19, a homologous RFX1 gene in *Caenorhabditis elegans* (*C. elegans*) is involved in ciliary neuron differentiation [18]. In humans, RFX1 is induced during retinoic acid (RA) mediated differentiation of hematopoietic stem cells [19] and in inner hair cell differentiation [20]. In cancer, RFX1 promotes differentiation of CSCs by directly and indirectly targeting major stemness regulators like c-Myc, FGF1, and CD44. Also, few drugs targeting pathways involving RFX1 are effective in reducing chemoresistance. RFX1, explicitly or implicitly, influence the significant pathways involved in cancer stemness, including Notch, Wnt, PIK3-AKT, and JNK/STAT. Finally, the altered levels of RFX1 have valuable prognostic value in breast cancer [21] and esophageal adenocarcinoma [3]. Our review aims to discuss the possibility of RFX1 targeted therapy in suitable cancers.

Current cancer treatments focus much on the cytotoxic capacity of chemical entities to eliminate cancer cells and have many shortcomings. Transcription factors were difficult to target without advanced knowledge of their structure, regulation, expression, and co-factors. From the current literature, we draw together different RFX1 transcriptional factor regulation systems to highlight the importance of its induction for targeted cancer therapy.

## Regulatory factor X1 (RFX1)

**Fig. 1:**
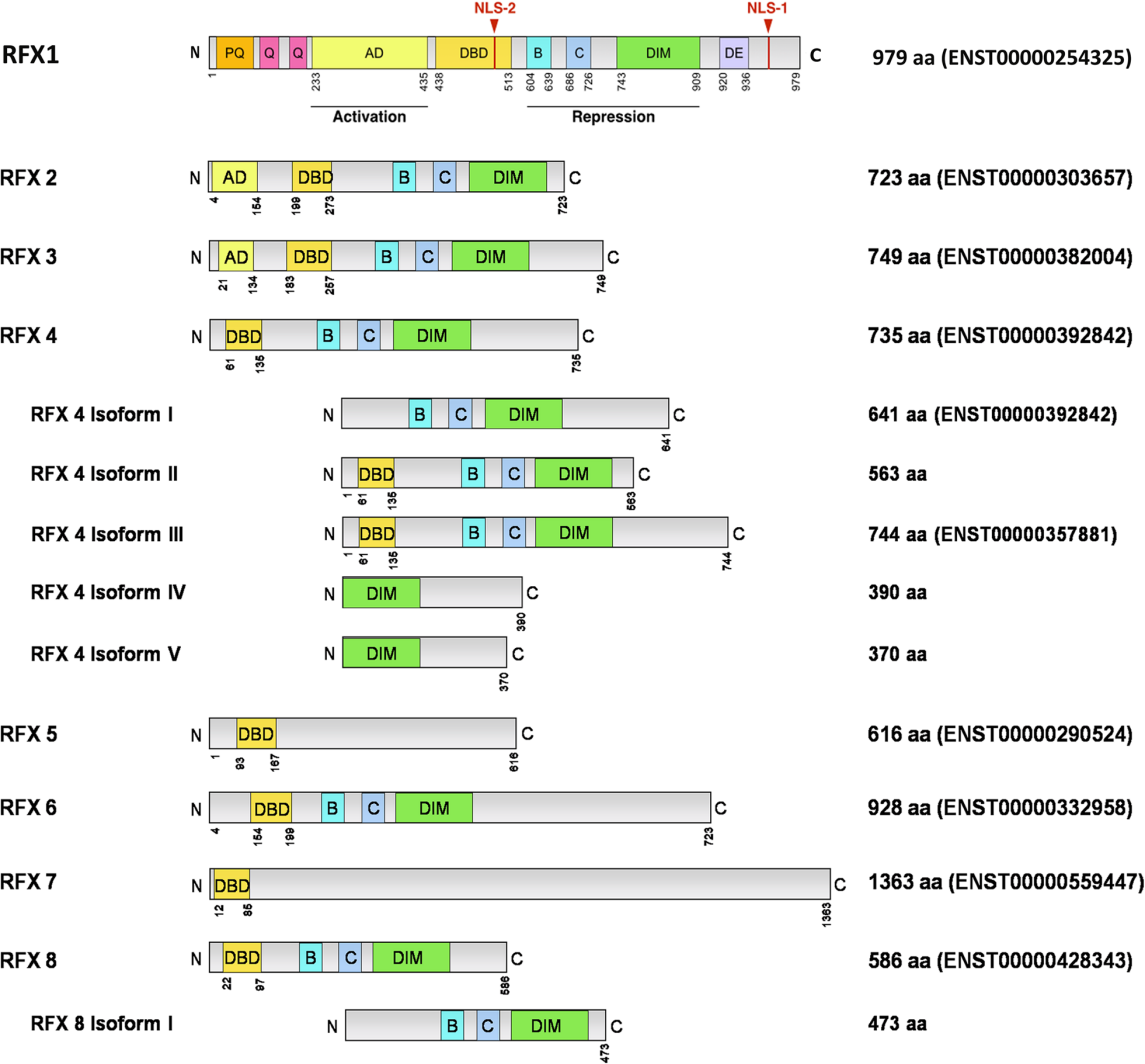
Structure of the human RFX transcription factors 1–8 with conserved DNA binding domains. Isoforms of RFX4 and RFX8 lack a DNB (DNA binding domain). DNB, B, C, and DIM (Dimerization domain) are conserved among most RFX proteins. RFX1 transcription factor has proline- and glutamine-rich region (PQ), glutamine-rich region (Q), glycine-rich region (G), a highly acidic stretch (DE) near the C-terminal region at 920–936 aa (amino acids), and nuclear localization signals (NLS-1 near c-terminal and NLS-2 within DBD)

RFX1 belongs to the family of regulatory factor X (RFX) transcription factors. RFX family of genes has a ubiquitous occurrence across the eukaryotes except for plants and algae [22]. RFX family proteins have an evolutionarily conserved winged-helix type DNA binding domain (DBD), found in a broad range of species from single-celled organisms like *Saccharomyces cerevisiae* (*S. cerevisiae*) to complex organisms like humans. *S. cerevisiae* and *C. elegans* have one RFX transcription factor each, namely Crt1 and Daf-19, respectively [23, 24]. Ciliogenesis and maintenance of cilia functions are the most studied functions of the RFX transcription factors. This function is conserved among invertebrates such as *C. elegans* [25] and *Drosophila melanogaster* (*D. melanogaster*) [26], as well as in vertebrates like zebra fish, frog, mouse [27, 28], and humans [29]. In humans, the RFX family constitutes eight genes, RFX1-8 [30] (Fig. 1). Transcript variants of RFX4 and RFX8 lacking a DBD were reported and possibly involved in different roles compared to other RFX proteins. Even though members of the RFX protein family such as RFX4-7 are linked to the formation or suppression of cancer [31–35], the broader regulatory role, defined activation and repression domains, and the unique mechanism of auto-repression make RFX1 an ideal candidate to be studied individually.

### Structure and functions

RFX1 was identified as a DNA binding protein that targets a particular X-box sequence in MHC class II genes. It has an evolutionarily conserved DBD, present at the core of RFX1 protein binds to the X-box motif present in target DNA. Apart from DBD, RFX1 and its isoforms share other conserved regions called B, C, and dimerization (DIM) domains [23]. Along with B and C domains, the DIM region forms the RFX1 homodimers or heterodimers [30, 36, 37]. Unlike many dimeric transcription factors, DNB and DIM in RFX1 are functionally distinct, and DIM is not involved in DNA binding, and they lie non-adjacent to each other [11, 30].

Moreover, RFX1 has a unique C-terminus extreme region compared to other RFX isoforms and a clearly defined glutamine-rich activator domain (AD) [38]. The pleiotropic behavior of RFX1 is attributed to the C-terminus repression domain and the N-terminus AD [39] (Fig. 1). For example, RFX1 induces Id2 gene transcription upon growth serum stimulation via its N-terminal while an N-terminal mutant with intact C-terminal promoted Id2 suppression [1]. Nuclear localization of RFX1 is mediated via the synergistic activity of a strong NLS present at the C-terminus extreme near the acidic region and a weak NLS within the DBD. The acidic stretch (DE) near the C-terminus seems to inhibit the binding of RFX1 to DNA inside the nucleus [37]. Unlike other helix-turn-helix proteins utilizing the helix structure, RFX1 utilizes a β-hairpin (called wing) structure to sense DNA [24].

In fungal species *Fusarium graminearum*, the role of RFX1 in DNA damage is well understood. In RFX1 deletion mutants, the double-stranded breaks in DNA appeared instinctively, leading to defective growth and development [40]. Other functions include its role as an effector in the DNA damage checkpoint pathway in *S. cerevisiae* [41], regulation of mitosis in *Schizosaccharomyces pombe* [42], and maintenance of synaptic functions in *D. melanogaster* [43].

Even though RFX1 is ubiquitously expressed in humans, the highest tissue-level expression of RFX1 is in the brain and testis. Though both are immune-privileged sites, any role of RFX1 in immune escape has not been discovered. Interestingly, a cell type-specific activity of RFX1 is observed in neuronal cells. RFX1 repressed the gene encoding Microtubule-associated protein (MAP1A) in non-neuronal cells but not in neuronal cells [44] and induces Alstrom syndrome gene (ALMS1) transcription in a context-dependent manner under serum-deprived growth arrest [45]. An RFX1 conditional knockout mouse had impaired testis cord development leading to inhibition of spermatogenesis and, hence was infertile [46]. The RFX1 homozygous knockout is embryonically lethal, highlighting the importance of functional RFX1 in embryonic development and survival [47]. Such studies signify the importance of yet under-explored and critical functions of RFX1. A relatively recent study illustrated that the RFX1/3 conditional knockout in mice brings about the loss of mature hair cells, fundamentally causing deafness [48]. With respect to the immune system, RFX1 acts as an activator of MHC Class II genes and represses MCP1, CD11a, CD70, IL17A, TLR4, and the TGFβ2 expressions, which could be instrumental in understanding etiology and materializing treatment strategies for cancer and other immune-related disorders.

Notably, RFX1 tends to be a negative regulator of oncogene expression in most cancers. Reportedly there are at least 10,000 binding sites of RFX1 in the human genome as conserved non-coding elements [49]. This implies the bigger picture that RFX1 plays in the human gene regulatory network. Moreover, using the affinity capture method and blotting analysis of mouse tissues, 79 binding sites of RFX1 were identified on 65 RNA binding protein (RBP) genes ascertaining the role of RFX1 in RNA processing and RNA editing [50]. A novel function of RFX1 was unveiled on high throughput screening of nucleosome-displacing factors (NDFs) in budding yeast. It was found to be a weak NDF, which on overexpression can switch to be a strong NDF [51]. These convincible traits make RFX1 an implicit target for the profound understanding of tumorigenesis and an immediate solution to current treatment shortcomings.

## RFX1 and cancer

**Fig.2:**
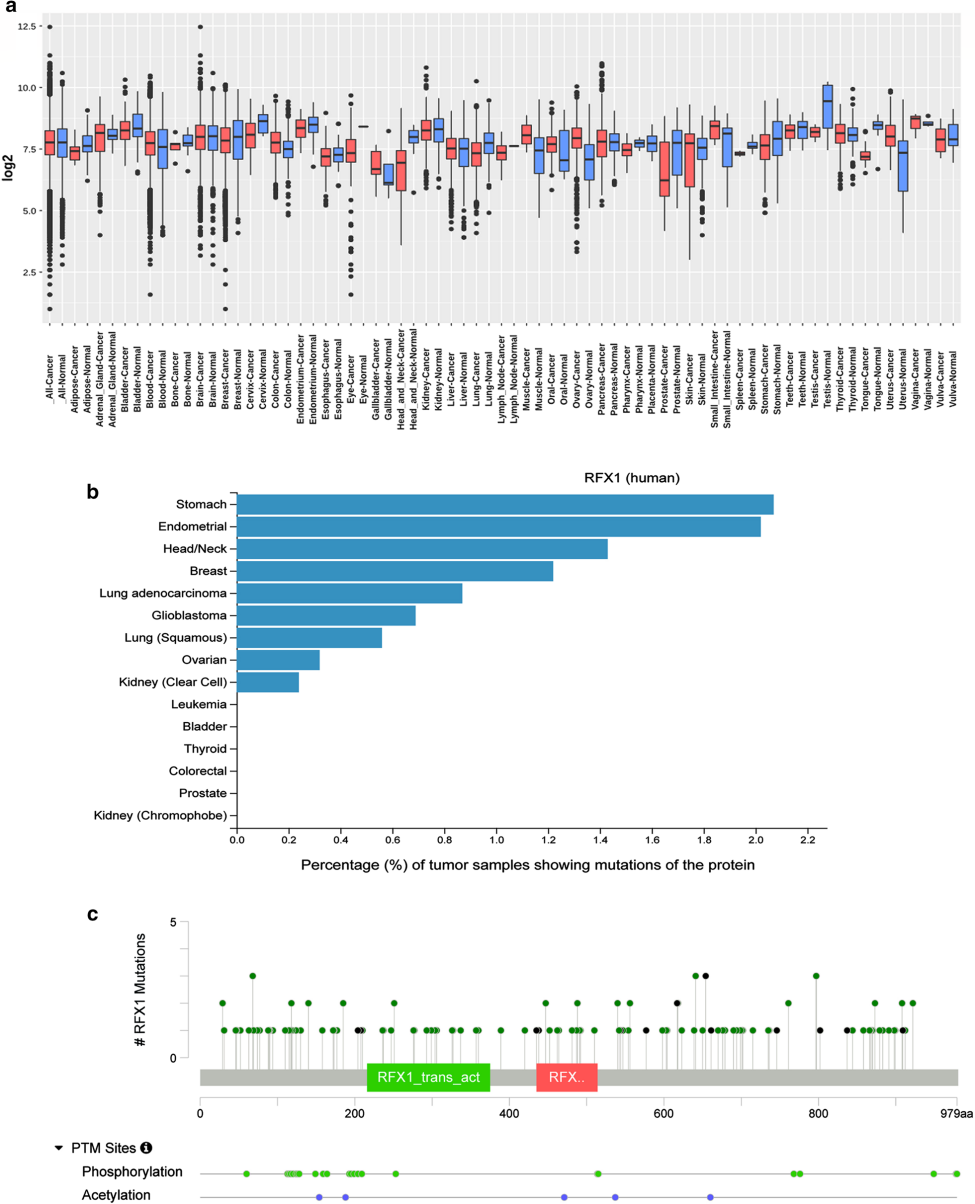
RFX1 expression and mutation analysis.** a** Boxplot graph illustrating the tissue-wide gene expression pattern of RFX1 across 70 paired tissues (GPL570) from micro-array database GENT2 (Access GENT2 free at http://gent2.appex.kr). Log2 fold changes of each cancer tissue compared to its normal tissues are plotted—Red (cancer samples) versus blue (normal samples). **b** This figure depicts the percentage of tumor samples with RFX1 missense mutations from 15 types of cancers on the TGCA database (https://www.phosphosite.org/proteinAction.action?id=5813) [55]. **c **The figure shows RFX1 mutations and PTM sites, visualized using cBioPortal, an open-source, public platform from a collective of 10,953 patients/10,967 samples comprising 46 different studies present in the TCGA Pan-Cancer Atlas Studies. The Lollipop plot illustrates a total of 111 missense mutations (green) and 21 truncating (black) mutations in 979 amino acids long RFX1 protein [150, 151]

Prevailing pieces of evidence suggest that RFX1 is a transcription factor that regulates a wide array of genes, and a better understanding of it's role can deduce its clinical implications in diseases like cancer. Tissue expression profiles from the GENT2 database exhibited a differential expression pattern of RFX1 in cancer tissues compared to normal tissues [52, 53] (Fig. 2a). A significant reduction of RFX1 expression in breast, bone, lung, oesophagus, prostate, and in 11 other cancer tissues were observed. At the same time, increased expression was observed in the ovary, pancreas, liver, and ten other tissues. Brain, kidney, and vulva cancer tissues did not show significant changes in RFX1 expression compared to normal tissues. Cancers with severe stages, grades, or subtypes associated with a poor prognosis, decreased survival and increased recurrence had decreased RFX1 expression. This trend was observed in colon, breast, blood, ovary, endometrial, and liver cancers as per the GENT2 database. For instance, RFX1 is least expressed in myeloma subsets of blood cancer, which has the least overall survival rate and reduced drug sensitivity [54]. The same goes for triple-negative breast cancer and poorly differentiated ovarian and colon cancers. Lung, brain, and kidney cancers did not have any observable correlation with RFX1 expression. Pancreatic and prostate cancers have an inverse correlation. The sample size was small in the case of the pancreas, thyroid, and adrenal gland cancers.

Another interesting correlation is that the tissues having decreased RFX1 expression also have the highest RFX1 missense mutation rate (Fig. 2b). The analysis considers 15 different cancer types with a total of 4440 TCGA tumor samples in the public repository PhosphoSitePlus [55]. For example, mutations at serine 117 (missense) and serine 204 (nonsense) sites of RFX1 have been observed in colon adenocarcinoma and bladder urothelial carcinoma, respectively (Fig. 2c). From these observations, it is clear that the altered expression and cancer types are crucial in interpreting RFX1 mediated gene regulation. Hence, both oncogenesis and tumor suppression events are to be discussed for evaluating the role of RFX1 in cancer.

### RFX1 in oncogenesis and tumor suppression

As mentioned earlier, RFX1 can regulate a handful of proto-oncogenes as well as tumor suppressors, eventually regulating cancer hallmarks. RFX1 hinders oncogenesis, being a transcriptional repressor of multiple oncogenes like c-Myc, FGF1, and other genes such as TGF-β2, COL1A1, COL1A2, CDX2, and TLR4. The shrinking levels of RFX1 expression during the tumorigenic transition of normal epithelia to adenocarcinoma in tissue samples attest to its anti-tumor activity [3]. RFX1 potentiates c-Abl kinase activity [56], the role of which varies among different cancers and its subtypes [57]. RFX1-mediated-tumor repression is also attained by inducing differentiation of cancer stem cells, thereby making cancer cells vulnerable to chemotherapy. RFX1 itself is a relevant tumor suppressor transcription factor in glioblastoma due to its negative regulation of CD44 expression [2]. On the contrary, weighted gene co-expression network analysis (WGCNA) of lung adenocarcinoma samples designated RFX1 as an enriched transcription factor in microtubule processing and tumor development, which needs further validation [58]. Recently, a dysregulated network of triplet molecules, including RFX1, TP73-AS1 lncRNA, and miR-197, was perceived to indicate a poor prognosis, survival, and tumor progression in glioblastoma multiforme [4].

### RFX1 regulates cancer cell epithelial-mesenchymal transition (EMT), invasion, and metastasis

EMT is a mechanism used by cancer cells to increase cells population having stemness nature to ensure the invasion and metastasis besides increased chemoresistance and survival [59]. A cohort study involving patients having small hepatocellular carcinoma (HCC) analyzed the use of RFX1 as a prognostic marker for this disease. The study revealed the down-regulation of RFX1 in cancer compared to healthy tissues as well as in patient samples. Overexpression of RFX1 decreased cancer invasion with decreased EMT marker, vimentin, and an increase in E-cadherin [60]. RFX1 down-regulated COL1A and COL1A2, which are potential markers of gastric cancer involved in extracellular matrix receptor interaction pathways [61–63]. DNA methyltransferase inhibitor, 5-aza-2′-deoxycytidine decreased RFX1/HDAC binding at COL1A2 transcription start site, proving RFX1 binding to the methylated DNA sequence [64]. A miRNA, miR-346 down-regulated RFX1 [8], and miR-346 inhibitor proved beneficial in reducing EMT in prostate cancer [65]. Phosphorylation of STAT3, induced by Interleukin 6 (IL-6), causes a deficiency in RFX1 mediated transcriptional regulation [15]. In breast cancer cell lines MDA-MB-468 and MCF-7, the IL-6/STA3 pathway plays a crucial role in EMT by upregulating TGF-β [66], a multifactorial cytokine that is again a target of RFX1 and a downstream signaling molecule of CD44 in breast cancer cells [67]. RFX1 also prevented the metastasis of multiple glioblastoma cell lines via down-regulating CD44 expression [2]. RFX1 knockout led to decreased HDAC1 and SUV39H1 recruitment to the MCP1 promoter, increasing its expression [68], which on the other hand, promotes EMT and cell migration in MCF-7 and OML1 (human head and neck cancer cell line) [8, 69]. RFX1 can inhibit cell migration and invasion by repressing FGF1 in many cancers [7, 70, 71]. Though in the majority of cases, RFX1 acts as a negative regulator of metastasis, it has been reported that the transcriptional activity of RFX1 was doubled in a metastatic ovarian cancer cell line HO-8910PM compared to its parent cell line, suggesting a positive role of RFX1 in cancer metastasis [72].

### RFX1 reduces chemoresistance and cancer recurrence

**Fig. 3:**
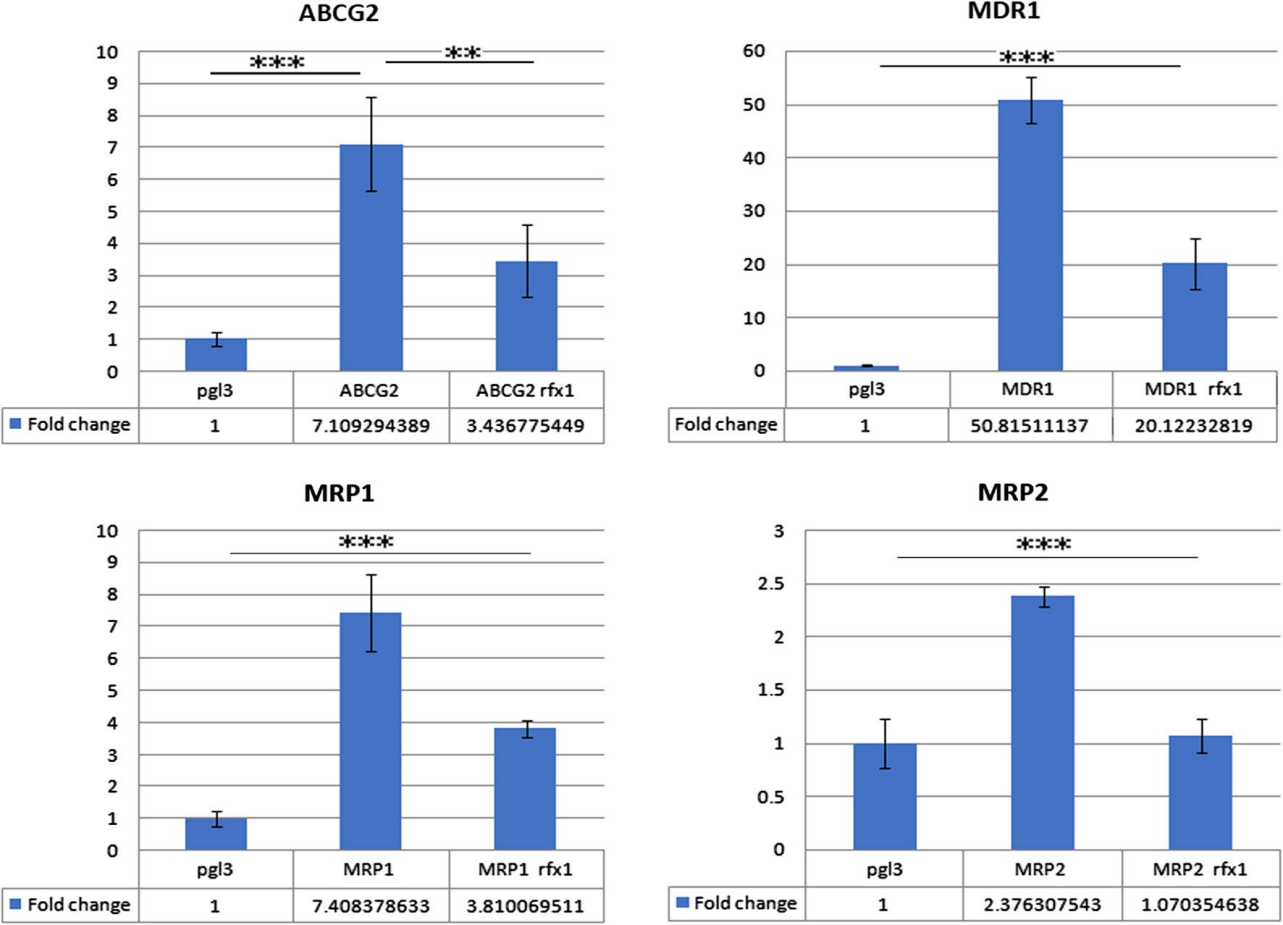
The figure depicts the luciferase activity of MDR promoters in control NT2 cells and on overexpression of RFX1. RFX1 downregulates the promoter activity of ABCG2, MDR1/ ABCB1, MRP1/ ABCC1, and MRP2/ ABCC2 ABC transporter proteins in NT2 cancer cell line (***p-value < 0.01; **p-value < 0.05; vertical line represent standard deviation)

The ability of CSCs to evade chemotherapy coupled with intrinsic property to form the bulk of tumors from a single clone could instigate cancer recurrence. The changes in RFX1 levels could play a significant role in attaining chemoresistance in cancer cells. The significant decrease in expression of RFX1 in HCC samples was correlated with increased chances of recurrence along with the arduous prognosis of early-stage HCC [60]. The role of RFX1 in chemoresistance was reassured when Sorafenib chemoresistance in HCC was effectively reversed using SC-2001, a small bipyrrole molecule that upregulates RFX1 [73]. Though direct evidence on RFX1-mediated chemo-sensitization is limited, RFX1 targets are often involved in mediating drug resistance and cancer recurrence. *In-silico* analysis of promoters of major MDR genes, including ABCB1, ABCC1, ABCC2, and ABCG2, predicted RFX1 as a common regulatory molecule on these promoters, denoting the possible transcriptional regulation of MDR genes by RFX1. Based on the works conducted in our lab on NTERA2 cl. D1 germ cell tumor cells, RFX1 down-regulated the promoter activity of MDR proteins involved in cancer stemness and drug resistance (Fig. 3) (unpublished results). COL1A1, already known to be negatively regulated by RFX1, is associated with decreased HCC patients' survival. Its down-regulation in HCC cell lines using siCOL1A impaired the tumorosphere formation in-vitro and down-regulated stemness markers like CD133 and OCT4 [74]. FGF1, a target that RFX1 negatively regulates, plays a significant role in conferring platinum drug resistance and ovarian cancer progression [75]. RFX1 down-regulation is also linked to TLR4 overexpression [14]. TLR4 overexpression promoted tumor metastasis, recurrence, and drug resistance in pancreatic and colorectal cancers [76–78]. Also, RFX1 can bind to the promoter of IL17A and negatively regulates its transcription. IL17A is involved in Cisplatin-resistance in colorectal cancer cell line HCT116 and is also known to promote chemoresistance in breast cancer patients [79]. These reports point out the crucial role of RFX1 in drug resistance and cancer recurrence. Despite these efforts, the functional role and mechanism by which RFX1 represses chemoresistance remain elusive. For this, proper elucidation of the RFX1 interactome coupled with the identification of key signalling pathways and identification of post-translational modifications of RFX1 and effect on DNA binding in different cancer types is necessary [80]. As particular inhibitors of transcriptions factors are difficult to synthesize, perturbation of protein partners involved in RFX1 regulation is key to understand RFX1 mediated chemosensitization pathways.

## RFX1 for cancer therapy

### Targeting RFX1 regulation

Cancer is a disease that manipulates major cellular regulation events to yield a suitable environment for its survival and spread. RFX1 being a major molecule regulating cellular integrity, demands the essentiality to look at its regulation and the deviations that result in a diseased state. Interventions targeted to regain proper regulation of RFX1 at epigenetic or transcriptional levels can have far-reaching outcomes and could be crucial in cancer treatment.

### Epigenetic regulation of the RFX1 gene

Cancer stem cell possesses a different epigenetic state compared to its stem cell counterpart. Epigenetic modifications are necessary to maintain the cancer stemness environment. Such variations are also seen in the case of RFX1. Ohashi et al*.* found that RFX1 is epigenetically silenced in human glioma tissue samples and cell lines compared to the healthy brain and lymphocyte counterparts [81]. Restriction landmark genomic scanning identified CpG hypermethylation at the seventh intronic region of the RFX1 enhancer. RFX1 expression was increased with methylation inhibitor 5- azacytidine, which reduced cancer cell proliferation [81]. Significant changes in the methylation pattern of RFX1 seventh intron were also observed in tissue samples of breast cancer patients compared to adjacent healthy tissues. Intriguingly, transcriptional regulation could be achieved at an intron farther downstream of a canonical promoter [82]. In a methylation pattern analysis of different subsets of glioma and normal brain tissues, RFX1 was hypermethylated in early-onset gliomas, secondary gliomas, astrocytic and oligodendroglial tumors. These tumors had increased expression of a methyltransferase enzyme enhancer of zeste human homolog 2 (EZH2) while having no alteration in insulin-like growth factor-binding protein 2 (IGFBP2) expression [83]. It is advised to determine the presence/absence of RFX1 promoter hypermethylation in other cancers and its relation with methyltransferases.

Another mechanism of RFX1 gene silencing was observed in the progression of systemic lupus erythematosus (SLE). When the seventh intronic region of the RFX1 enhancer is unmethylated, IL-6 induces the phosphorylation of STAT3, which then binds to this region to repress RFX1 expression in CD4^+^ T cells. RFX1 down-regulation continued to promote unchecked IL17A expression [15]. The reversal of RFX1 gene silencing at the epigenetic level is a promising strategy for treating cancers. RFX1 epigenetics is in its infancy and needs meticulous inspection. Lysyl oxidase-like 3 (LOXL3) has a proximal 5′-UTR site without a typical TATA box and CAAT box, suggesting a different way of regulation with putative binding sites for MIBP/RFX1 and its interacting partners like Nuclear factor one (NF1) and cAMP responsive element binding protein 1 (CREB). RFX1 also has a putative binding site at the human kinesin family member 3A (KIF3A) gene promoter, whose inhibition contributed to the suppression of an aggressive form of breast cancer [84, 85].

### Transcriptional and Post-transcriptional regulation

One of the most understood ways of regulating RFX1′s gene expression is by its autorepression RFX1 auto-represses itself with the assistance of yet unidentified co-repressor, in response to DNA replication arrest, to bring about a controlled expression of RFX1 [86]. Interestingly, the RFX1-mediated auto-repression could be partly due to the direct interaction of RFX1 with serine/threonine protein phosphatase catalytic subunit (PP1c). The RFX1-PP1c complex binds to the RFX1 promoter region, suggesting the role of RFX1 to recruit PP1c for its negative regulation [87]. Recently, Festin (a natural flavonoid PP1 inhibitor) successfully sensitized HDACis-R (histone deacetylase inhibitors-resistant) HCC cell line to HDAC inhibitors. A PP1 activator, in turn, increased drug resistance in HDACis-R cells. Experimental evidence for transcriptional regulation of RFX1 is rare and limited [88]. RFX1 downregulated cancers may benefit from targeting uncontrolled autorepression of RFX1.

Some key miRNAs and lncRNAs affecting RFX1 could be instrumental in its regulation in cancer. TargetScan Human application (version 7.2 March 2018) predicted highly conserved 18 miRNAs (some of which are hsa-miR-124-3p.1, hsa-miR-506-3p, hsa-miR-19a-3p, hsa-miR-92a-3p, hsa-miR-367-3p, hsa-miR-200a-3p, hsa-miR-141-3p, hsa-miR-300, hsa-miR-381-3p, hsa-miR-140-3p.2) and poorly conserved 593 miRNAs among vertebrates targeting 1173 base pairs long RFX1 3′UTR [89]. Some of these miRNAs hold promise in the treatment against cancer.

A miRNA, miR-346, whose level was significantly high after Leishmania infection, can down-regulate RFX1 [8]. miR-346 has been known to promote cell proliferation and decrease apoptosis in C42 and LNCaP prostate cancer cell lines [90]. Another study revealed that 3′UTR of RFX1 is targeted by miR-92, and the resultant RFX1 down-regulation could be the reason for the up-regulation of a renowned proliferation marker, PCNA [91].

The aforementioned WGCNA analysis also reported RFX1 as the target of miR-184, a miRNA that is differentially expressed in lung adenocarcinoma [58]. Previous studies indicated that miR-184 was down-regulated in nasopharyngeal carcinoma and could act as a tumor suppressor [92] while being upregulated in multiple HCC cell lines like Hep3B, BEL-7402, MHCC97H, HCCC-9810, MHCC97L, and Huh7 [93, 94]. In a relatively recent study, RA-induced RFX1 acts as a transcription factor for increased miR29c expression in primary goose hepatocytes [95]. miR29c downregulation is witnessed in the progression of gastric cancer [96], HCC [97], gliomas [98], and chronic lymphocytic leukemia [99]. Further, miR29c promotes cancer stem cell differentiation and apoptosis, as evident in P19 teratocarcinoma cells [100].

### Post-translational regulation

A mechanism involving RFX1 degradation was noted in the origin of SLE. In SLE cases, an E3 ligase, STIP1 homology, and U-box containing protein 1 (STUB1) help in the proteasomal degradation of RFX1, following its polyubiquitination modification [101]. The existence of a similar mechanism in cancer is yet to be determined. Another possible ubiquitination site was identified at the lysine 537 of RFX1 protein using an anti-di-glycine antibody and mass spectrometry [102]. In a study involving murine hypothalamic neuronal cells N1, RFX1 phosphorylation by Glycogen synthase kinase 3 beta (GSK3β) inhibited the RFX1-mediated transcriptional regulation. Site-directed mutagenesis of GSK3β sites of RFX1 confirmed serine residue at 120 and threonine at 124 to be phosphorylated in human bone marrow neuroblast cell line SH-SY5Y [85]. GSK3 inhibitors have been promising agents in treating cancers and other illnesses, including diabetes, inflammation, and neurodegeneration [103]. Again, the roles of these modifications in cancers are yet to be explored.

### Co-activators and co-repressors

**Fig. 4:**
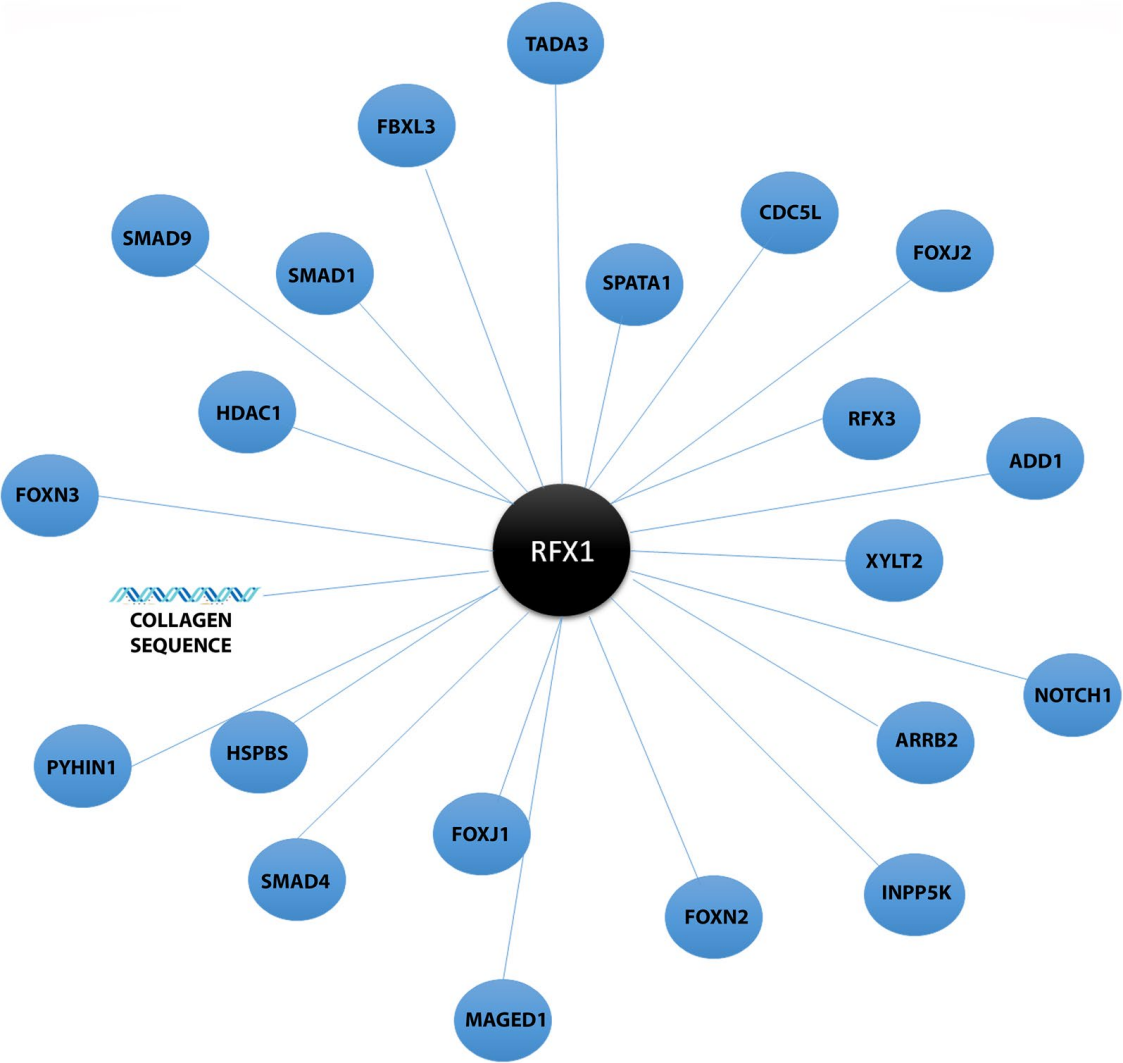
Protein–protein and protein-DNA interactions of human RFX1 protein curated using InAct database and analysis tool [152]

Co-regulators play a key role in dictating the function of a target molecule (Table 2). This is especially relevant in the case of RFX1, as seen in HL60 cancer cells. Even though RFX1 is ubiquitously expressed in undifferentiated HL60 cells, its ability to act as a repressor or activator relies on the co-partner Myc intron-binding protein 1 (MIBP1). An interactome map omitting linker genes constructed from different breast cancer types identified RFX1 as a differentially expressed molecule interacting with HER2 in HER2 positive breast cancer tissues. RFX1 was significantly down-regulated in HER2 positive cancer tissues compared to other subtypes and may aid in targeted therapy and Lapatinib sensitization [122]. RFX1 forms dimers with RFX2 and RFX3 proteins, and it is assumed that these proteins may target overlapping target genes [16]. Hence, deciphering the RFX1 dimerization patterns and followed-up mode of regulations are fundamental for a deeper understanding of RFX1-mediated regulation. Future studies are required to demystify the varying roles of a vast majority of RFX1 interactions (Fig. 4).

**Table 2:** RFX1 co-activators and co-repressor

RFX1 interacting protein	Co-activator	Co-repressor	Function
MIBP1			Nuclear localization of RFX1; transcriptional repression of c-Myc in RA induced HL60 cell line [19]
			Upregulation of MHC class II gene transcription [11, 12] transactivation of HBV enhancer activity [36]
AP4			Increase SHP1 transcription in MCF-7 cell lines to reduce cell proliferation [81]
NF1			NF1/RFX1 binds to P sequence element of chorionic somatomammotropins gene promoter and represses its activity [162]
ADD1			Co-repressor of RFX1 in the COS-7 kidney fibroblast cell line [163]
p107			p107/ RFX1 utilized the RFX1 binding site and repressed PCNA [157]
CREB			CCAR2 stabilizes RFX1/CREB complex which transcriptionally regulates cell cycle genes involved in cancer progression in HO1N1 SCC cell line [144]
PP1c			Involved in RFX1 autorepression [87]
HDAC			RFX1 recruits HDAC and represses COL1A2 promoter activity [64]
mSin3A			RFX1 recruits mSin3A and represses COL1A2 promoter activity [64]

*AP4* adaptor protein 4, *ADD1* Alpha adducin, *SCC* squamous cell carcinoma

### RFX1 could target CSCs by inducing differentiation and targeting key genes

The role of RFX1 in the differentiation of stem cells can be fully apprehended by looking at some examples of normal stem cell development. Transcriptome analysis of porcine bone mesenchymal stem cell and adipose mesenchymal stem cells (ASC) identified RFX1 as an essential transcription factor involved in their differentiation [104]. RFX1 is a crucial transcription factor for the activation of Interleukin-5 Receptor Alpha (IL5RA), which in turn dictates the late differentiation of myeloid progenitors [16]. In another study, RFX1 induced differentiation of the inner hair cells from patient-specific pluripotent stem cells [20].

In tumor physiology, unregulated Myc expression obstructs cancer cell differentiation, as seen in the mouse erythroleukaemia cell [105]. c-Myc induces stem cell-like characteristics in both colon and colorectal cancers [106, 107]. Myc gene, along with Ras oncogene, successfully catalyzed tumor conversion of primary embryo fibroblasts [108]. Interestingly, RFX1 can modulate both c-Myc transcriptional elongation and Ras-induced transcription [109]. In the case of undifferentiated HL60 myeloid leukemia cell line, a molecule named phorbol 12-myristate 13-acetate (PMA) was able to increase nuclear localization of RFX1, which in turn bound to the x-box region in the myc gene and repressed its transcription [110]. PMA can induce protein kinase C (PKC) activity [111] and is well known for its ability to induce differentiation of monocytes to macrophages [112]. PKC plays a key role in differentiation mediated by PMA as well as retinoic acid [113]. In undifferentiated HL60 cells, upon PMA treatment, PKC increases nuclear translocation of RFX1 for c-Myc repression, making RFX1 a possible factor involved in the differentiation process. The induction of differentiation by RFX1, which helps in the inhibition of cancer stemness, is a promising therapeutic intervention and could prevent recurrence and resistance.

In a study, overexpression of RFX1 reduced FGF-1B mRNA expression, and neurosphere formation resulted in the inhibition of self-renewal in glioblastoma stem cells [7]. A similar study using glioblastoma cell lines proved that RFX1 overexpression led to decreased cell invasion, migration, and proliferation mediated by direct targeting of CD44 [2]. Also, overexpression of RFX1 using lentiviral transfection in the F98 glioblastoma cell line caused a marked reduction in cell proliferation [114]. SC-2001 boosted RFX1 expression in HCC and induced cellular apoptosis with better anti-tumor activity than Sorafenib, an FDA-approved medication for HCC [73]. This activity was attributed to SHP1 aided the nuclear localization of RFX1. Down-regulation of RFX1 was correlated with decreased H3K9 tri-methylation and increased expression of CD70 and CD11a in SLE [115]. CD70^+^ population is useful in isolating CSCs from breast cancer cell lines, and CD70^+^ CSCs had significant self-renewal capacity and promoted lung-metastasis [116].

### RFX1 targets key signaling pathways associated with the survival and maintenance of CSCs

Some of the significant pathways that CSCs utilize to promote stemness, evade treatment, and proliferate recklessly are Wnt, Notch, Hedgehog, TGF-β, PI3K/AKT JNK/STAT signaling pathways. As discussed, increased RFX1 expression was associated with CD44 down-regulation in glioblastoma. CD44, when active, induces Wnt/β-catenin signaling in colorectal cancers [117]. Another RFX1 target, c-Myc, is a downstream molecule in the Wnt pathway. Down-regulation of Wnt/β-catenin in P19 teratocarcinoma cells resulted in reduced levels of c-Myc, which in turn induced differentiation and inhibited proliferation [118]. CD70, a positive regulator of the Wnt pathway, promotes drug-resistance in leukemia CSCs even after Imatinib treatment. Imatinib is an inhibitor of BCR–ABL1 fusion in chronic myeloid leukemia (CML). The inhibition of CD70, along with Imatinib treatment, effectively eliminated CD34^+^ chronic myeloid leukemia (CML) stem cells in-vitro [119].

Upregulated Notch signaling in many cancers is related to resistance, recurrence, and the disease's poor prognosis [120–124]. There are many ways by which Notch signaling gets activated in cancer cells, and one of the Notch inducers is FGF1 [121], which RFX1 negatively regulates. FGF1 increases matrix metalloproteinase-9 (MMP-9) in ENU1564 breast cancer via activating PI3K/AKT pathway, eventually promoting cancer metastasis [125]. RFX1 is also reported to interact with an intracellular active form of NOTCH-1 (ICN1) in human T-cell acute lymphoblastic leukemia (T-ALL) cells [126]. ICN1 overexpression, along with constitutive NOTCH-1 activation, is associated with T-ALL occurrences [127]. NOTCH-1can increase c-Myc expression leading to tumorigenesis in T-ALL [128]. RFX1 is known to down-regulate c-Myc transcription. Exploring the regulatory role of RFX1 as a DNA binding partner of ICN1 can help comprehend T-ALL etiology and treatment. CDX2, a NOTCH-1regulated gene involved in Barret's adenocarcinoma progression, is negatively regulated by RFX1 [129]. In hypoxic adenocarcinoma of the lung (ACL) cell lines, NOTCH-1activates Akt for increased cancer cells' survival. This strong activation is a result of NOTCH-1 mediated up-regulation of IGF-1R [130]. RFX1 is a key player in the suppression of IGF-1 mediated breast cancer cell proliferation [81]. IGF-1 is a pro-survival factor and is ill-used by cancer cells to prevent apoptosis by activating PI3K/AKT signaling [131, 132]. SHP1, with the help of RFX1, could inhibit IGF1-induced cell proliferation, thereby accentuating survival in breast cancer cell lines MCF-7 and ZR-75–1. IGF1-induced cell proliferation by MEK/Erk pathway is countered by the c-Jun N-terminal kinase (JNK) pathway, activating SHP1 promoter by transcription factors, RFX1 and AP4 [81].

STAT3 phosphorylation and subsequent activation can effectively induce STAT3 mediated epigenetic silencing of RFX1. STAT3 targeted therapy holds potential as its key to the maintenance of several hallmarks of CSCs [133–135]. RFX1 inhibits the SHP1/STAT3 pathway [73] and the SHP1/STAT3/MCL1 axis [10], contributing to improved therapeutic outcomes in HCC. The role of RFX1 in the IL-6/STAT3 pathway observed in SLE has already been addressed earlier. RFX1 also suppresses MCP1 transcription, which is known to induce EMT in head and neck cancers through AKT/STAT3/SNAIL signaling [8]. Another cytokine, IL17A, a downstream target of STAT3, is a key molecule in the production of IL-6 [135] and is also negatively regulated by RFX1 [15]. Cross talk between nitric oxide-induced Notch causes the constitutive expression of IL-6 induced STAT3 is seen vital for breast cancer stemness paves the way to identify targets common to both pathways [136].

TGF-β is known to function in SMAD-dependent and independent pathways [137]. RFX1 could affect both sets of pathways as RFX1 is known to interact with SMAD1 and SMAD4 [138]. It can also act on the TGF-β2 receptor and down-regulate it resulting in decreased activation of phosphorylated ERK, thereby affecting TGF-β/MEK/ERK pathway [139]. These activities could have a possible tumor-suppressive activity. It is interesting to note that a TGF-β2 inhibitor could force dormant cancer cells to come out of dormancy [140]. TGF-β is known to induce both COL1A1 and COL1A2 expression in human fibroblasts [134].

## Conclusions and perspectives

**Fig. 5:**
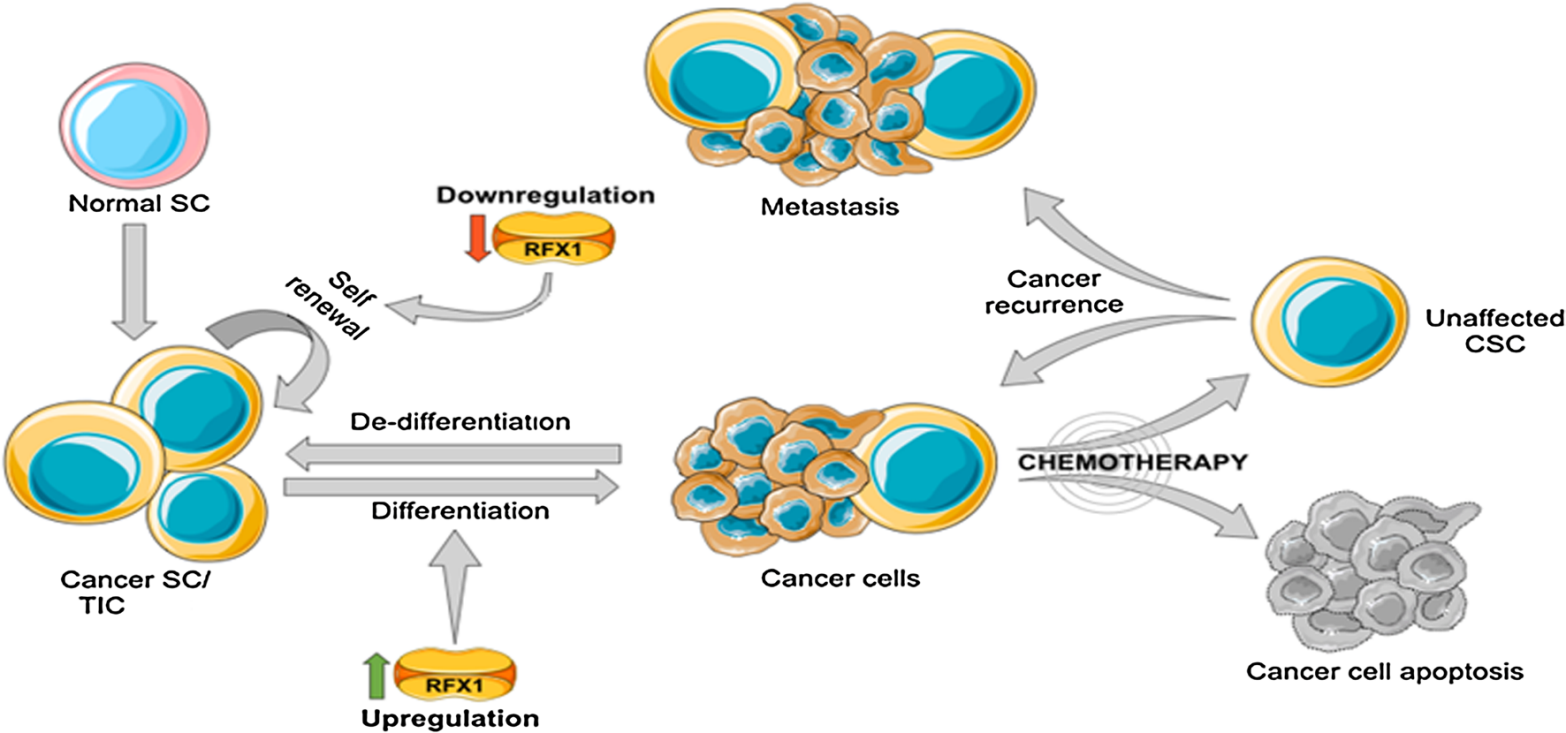
RFX1 operates as a key molecule targeting cancer stem cells

Current cancer treatments revolve around a combination of drugs rather than relying on an individual drug. There have always been attempts to find the 'key molecule' involved in carcinogenesis, but interventions targeting any such molecule have been complicated and had limited success. Even though classical drugs effectively target cancer cells, most do not prevent cancer recurrence due to their inability to target unique CSC populations. On the other hand, combinational therapy had more success compared to the use of single drugs. This strategy has excellent potential when a combination of drugs targeting CSCs, and other targeting differentiated cancer cells are administered together [141]. SC-2001, an RFX1 inducing agent when coupled with Sorafenib, effectively reversed chemoresistance in a xenograft mice model of Sorafenib-resistant HCC [73]. One mechanism by which these CSCs-targeting drugs act is by converting malignant tumor stem cell populations to differentiated cells, which are then susceptible to classical drugs [142]. Transcription factors are the key to such reprogramming [143]. RFX1-mediated targeting provides one more arsenal targeting the resilient CSC populations (Fig. 5).

In conclusion, we propose that RFX1 is a promising candidate that could bridge the gap we currently have in cancer treatment. Inducing RFX1 expression yielded positive results in cancers such as gastric cancer [2], breast cancer [21], glioma [2], esophageal adenocarcinoma [3], and HCC [10]. Simultaneously, the treatment of HCC, SCC, and ovarian cancers should be under caution. For instance, in a p53 independent SCC system, CCAR2 stabilized the RFX1 CREB-1 association to promote tumorigenesis [144]. This may be linked to the failure of SIRT1 to deacetylate RFX1 in this particular case. It is unknown what kind of regulation RFX1 exerts in the presence of intact or mutated p53. It would not be ideal for targeting SCC using RFX1 as a target until a clear regulation pathway is known. In HCC, 80% of cases are due to manifestations of Hepatitis infection (both HBV and HCV) [145], and RFX1 is known to reactivate Hepatitis B virus infection induced by doxorubicin treatment in HCC cell lines [146]. However, it is interesting to note that RFX1 upregulation was HBV EP element mediated, and RFX1 shows its pleiotropic role at EP element. p53 is a repressor of HBV transcription only in the presence of an RFX1 bound intact EP element, and a mutation of EP element makes p53 an activator of HBV transcription in HCC cells [147].

Taken altogether, RFX1 is a tumor suppressor protein having a broad impact on multiple genes involved in oncogenesis and tumor suppression, collectively favoring a suitable niche for anti-cancer battle in particular cancers. From our current understanding, it is clear that RFX1 can target CSCs in multiple cancer subtypes and be an implicit arsenal against the tumor, alone or in combination. Still, the dual nature of RFX1 demands the usage of targeted therapy than a broad induction as in the case of many promising transcription factors in cancer therapy. As novel techniques like proteolysis targeting chimaeras (PROTACs) and nanotechnology develop, targeted therapy of transcriptions factors could become a reality [148]. Transcription factors can also be targeted indirectly by modulating histone modifications and protein–protein interactions. Protein phosphatase 1 (PP1c) acts as a co-repressor and represses RFX1 targets in an HDAC independent manner [87], which could limit the effects of RFX1 regulation to few genes [149]. Besides that, before strategizing RFX1 as an arsenal against cancer progression, certain key mechanisms need further exploration including the role of its interacting partners and epigenetic modifications which further dictate the pleiotropic behavior of RFX1. A direct role of RFX1 in modulating crucial signalling pathways involved in cancer is limited and deciphering such pathways could shed light on ‘how RFX1 affects chemoresistance?’ Other areas which need further attention are the regulation of RFX1 enhancer activity, miRNA regulation of RFX1 in cancers, and its role in RNA processing and editing as seen in mouse tissues [50].

As per the current scenario, cancer drug resistance is one of the major drawbacks of the current chemotherapy regimen, and combinations of drugs have successfully overcome resistance to some extent. RFX1 is a multi-disciplinary regulatory molecule that can interact with RNA, DNA, and proteins, thereby playing distinctive roles in epigenetic modifications, RNA processing, cell cycle regulation, nuclear transport, transcriptional regulation, cell signaling, immune regulation, and so on.

## Data Availability

Not applicable.
